# Urinary Metabolite Diagnostic and Prognostic Liquid Biopsy Biomarkers of Lung Cancer in Nonsmokers and Tobacco Smokers

**DOI:** 10.1158/1078-0432.CCR-24-0637

**Published:** 2024-06-05

**Authors:** Bhavik Dalal, Takeshi Tada, Daxesh P. Patel, Sharon R. Pine, Mohammed Khan, Takahiro Oike, Yasuyuki Kanke, Amelia L. Parker, Majda Haznadar, Leila Toulabi, Kristopher W. Krausz, Ana I. Robles, Elise D. Bowman, Frank J. Gonzalez, Curtis C. Harris

**Affiliations:** 1 Laboratory of Human Carcinogenesis, Center for Cancer Research, National Cancer Institute, Bethesda, Maryland.; 2 Division of Medical Oncology, Department of Medicine, School of Medicine, University of Colorado Anschutz Medical Campus, Aurora, Colorado.; 3 Laboratory of Metabolism, Center for Cancer Research, National Cancer Institute, Bethesda, Maryland.; 4 Office of Cancer Clinical Proteomics Research, Division of Cancer Treatment and Diagnosis, National Cancer Institute, Rockville, Maryland.

## Abstract

**Purpose:**

Nonsmokers account for 10% to 13% of all lung cancer cases in the United States. Etiology is attributed to multiple risk factors including exposure to secondhand smoking, asbestos, environmental pollution, and radon, but these exposures are not within the current eligibility criteria for early lung cancer screening by low-dose CT (LDCT).

**Experimental Design:**

Urine samples were collected from two independent cohorts comprising 846 participants (exploratory cohort) and 505 participants (validation cohort). The cancer urinary biomarkers, creatine riboside (CR) and N-acetylneuraminic acid (NANA), were analyzed and quantified using liquid chromatography–mass spectrometry to determine if nonsmoker cases can be distinguished from sex and age-matched controls in comparison with tobacco smoker cases and controls, potentially leading to more precise eligibility criteria for LDCT screening.

**Results:**

Urinary levels of CR and NANA were significantly higher and comparable in nonsmokers and tobacco smoker cases than population controls in both cohorts. Receiver operating characteristic analysis for combined CR and NANA levels in nonsmokers of the exploratory cohort resulted in better predictive performance with the AUC of 0.94, whereas the validation cohort nonsmokers had an AUC of 0.80. Kaplan–Meier survival curves showed that high levels of CR and NANA were associated with increased cancer-specific death in nonsmokers as well as tobacco smoker cases in both cohorts.

**Conclusions:**

Measuring CR and NANA in urine liquid biopsies could identify nonsmokers at high risk for lung cancer as candidates for LDCT screening and warrant prospective studies of these biomarkers.

**Translational Relevance**

Every year, 20,000 to 40,000 people who do not smoke develop lung cancer. The total number and proportion of nonsmoker lung cancer fatalities in the United States have climbed from 1964 to 2015 and are predicted to increase during the following few decades until 2065. Identifying nonsmokers at high risk of getting lung cancer is critical because they are currently ineligible for government-funded low-dose CT screening. In this study, we demonstrate that creatine riboside and N-acetylneuraminic acid (NANA) are significantly higher and comparable in nonsmoker and tobacco smoker cases compared with population controls in two independent cohorts. Furthermore, these cancer urinary biomarkers are associated with increased cancer-specific death in both nonsmoker and tobacco smoker cases. This liquid biopsy should be a low-cost, noninvasive, and high-throughput technique to achieve cancer detection and indicate the need for therapy in early-stage nonsmoker cases to improve lung cancer survival outcomes.

## Introduction

Lung cancer is the most common type of cancer and the main cause of cancer deaths globally (1). The prognosis is poor with 5-year survival rates of 61.2% for localized tumors and only 7.0% for distant tumors (2). However, these survival rates might be improved by establishing rapid and low-cost biomarker prescreening to assist in the precise and credible diagnosis and prognosis of lung cancer. The US-based National Lung Screening Trial first showed reduced lung cancer mortality from low-dose CT (LDCT) screening for individuals at a high risk of developing lung cancer when compared with chest X-ray (3). In Europe, several trials also revealed that LDCT screening reduces lung cancer mortality (4–6). Based on the results of these trials, LDCT screening has been recommended to detect lung cancer in high-risk populations (7, 8). The US Preventive Services Task Force recommends annual screening for lung cancer with LDCT in adults aged 50 to 80 years who have a 20-pack-year smoking history and currently smoke or have quit within the past 15 years (9). However, this excludes many individuals at high risk of having lung cancer who are not eligible for screening. Moreover, current LDCT screening leads to high false-positive rates (10), which causes unnecessary and potentially harmful diagnostic procedures, including surgical resections, that may incur anxiety, economic burden, and increased mortality for patients (3). Liquid biopsy-derived biomarkers have demonstrated encouraging results in the early identification of lung cancer (11), and therefore, it would be of great value to develop complementary and accurate biomarkers that can facilitate the further identification of high-risk individuals.

Risk factors for lung cancer in a person who does not smoke (nonsmoker) include exposure to radon, asbestos, coal, and secondhand smoke (SHS) and being female or of Asian descent, among others (12–15). An estimated 10% to 15% of lung cancer cases in Europe and North America occur in nonsmokers (16, 17). Lung cancer in nonsmokers has been reported to be higher in Asia, where it reaches nearly 40% of all lung cancer cases (18). The proportion of patients with non–small cell lung cancer (NSCLC) who do not smoke in the United States was reported to be increasing to 14.9% in 2011 to 2013 from 8% in 1990 to 1995 (19). The absolute number and proportion of individuals and the number and proportion of lung cancer deaths in nonsmoker individuals in the United States have increased from 1964 to 2015 and are modeled to continue increasing over the next several decades (20). Identifying nonsmoker individuals who are at high risk for developing lung cancer is, therefore, of paramount importance as they currently are not eligible to receive LDCT screening according to the US Preventive Services Task Force recommendations (7, 9, 21). An International Association for the Study of Lung Cancer Early Detection and Screening Committee Report recommended lung cancer screening and the development of an accurate risk prediction model for nonsmoker individuals (20). The latest study reported that the conventional screening criteria miss 50% of patients with lung cancer, and that age and smoking history may not be sufficient predictors of lung cancer risk and prognosis (22). Additionally, a recent study developed models that can accurately identify lung cancer in a high-risk population, regardless of smoking status (23).

We previously conducted metabolomic profiling of urine samples collected from 1,005 participants using ultraperformance liquid chromatography coupled with tandem mass spectrometry (UPLC–MS/MS) and found that creatine riboside (CR) and *N*-acetylneuraminic acid (NANA) levels are significantly elevated in stages I and II patients with NSCLC compared with population controls (24). In addition, we found that CR and NANA levels are increased in tumor tissues compared with matched nonadjacent tissues of patients with NSCLC, with levels of metabolites positively correlated between matched tissue and urine (24). That study largely comprised cases of smoking exposure. Here, we hypothesized that CR and NANA may also be robust diagnostic indicators of lung cancer in nonsmokers, and we utilized lung cancer specimens from two independent cohorts to identify patients at high risk of recurrence.

## Materials and Methods

### Study design

The exploratory cohort included urine samples from 375 patients with NSCLC and 471 population controls comprising 529 people with active tobacco use (smokers) and 317 nonsmokers from the greater Baltimore, MD, area in the period between August 1997 and June 2019. The validation cohort included urine samples from 285 patients with NSCLC and 220 population controls comprising 315 smokers and 190 who were nonsmokers. For the validation cohort, each urine sample was collected in a 60 mL specimen cup and refrigerated at −80°C until use. For the exploratory cohort, details were described previously (24). Population controls were identified from the Department of Motor Vehicles lists and frequency matched to cases based on age, sex, and self-reported race. We preferred to include population controls 60 years of age and older and attempted to have a balanced distribution of males and females. Population controls who developed cancers during the next 5 years after recruitment into the study were excluded. Cancer cases were not diagnosed with cancer types other than lung cancer. Each urine sample was collected in a sterile 50 mL container and delivered to the University of Maryland, where it was divided into 10 mL aliquots and refrigerated at −80°C until use. Urine samples were thawed on wet ice at the time of use. For this present study, no formal statistical power calculations were applied to estimate sample size. Survival times were calculated from the time of diagnosis to the time of death or censor; death related to lung cancer was determined using the National Death Index extraction of the death certificates (25). A pathologist performed tumor staging using the seventh edition of the AJCC’s Cancer Staging Manual (26). The questionnaire data were used to obtain information on parental childhood secondhand smoking exposure. The following information was gathered on whether parents have smoked in the childhood or adulthood home; whether the mother, father, or both have smoked in the childhood or adulthood home; whether the parents smoked lightly, moderately, or heavily; the average number of cigarettes smoked; and how many years the parents smoked in the childhood or adulthood home. Secondhand smoke includes childhood, adulthood, and work exposure. Details about the clinical and demographic characteristics of the subjects included in the study are presented in Table 1. Written informed consent or waiver of consent was obtained for experimentation with all enrolled participants. The exploratory cohort study was approved by the appropriate Institutional Review Boards and conducted in accordance with the Declaration of Helsinki. The validation cohort study was approved by the University of Colorado Cancer Center PRMS Expedited Review Committee (PRMS 23-110) and the Colorado Multiple Institutional Review Board (23-0941).

**Table 1.** Clinical characteristics of the study participants by smoking status.

Exploratory cohort	Lung cancer cases		Population controls	
*N* = 846
Clinical characteristics
	Smoker	Nonsmoker	Smoker	Nonsmoker
Participants, *n* (%)	261 (70)	114 (30)	268 (57)	203 (43)
Age, mean ± SD	66 ± 6	65 ± 14	66 ± 9	67 ± 9
Race, *n* (%)				
European American	192 (64)	110 (36)	151 (59)	106 (41)
African American	69 (94)	4 (6)	117 (55)	97 (45)
Gender, *n* (%)				
Male	147 (81)	34 (19)	153 (64)	86 (36)
Female	114 (59)	80 (41)	115 (50)	117 (50)
SHS exposure, *n* (%)	250 (49)	95 (35)	256 (51)	176 (65)
Histology, *n* (%)				
Adenocarcinoma	163 (61)	104 (39)		
Squamous cell carcinoma	98 (91)	10 (9)		
Stage, *n* (%)				
1A	73 (75)	24 (25)		
1B	66 (86)	11 (14)		
2A	12 (67)	6 (33)		
2B	20 (74)	7 (26)		
3A	27 (71)	11 (29)		
3B	19 (54)	16 (46)		
4	27 (47)	31 (53)		

Validation cohort	Lung cancer cases		Population controls	
*N* = 505
Clinical characteristics	Smoker	Nonsmoker	Smoker	Nonsmoker
Participants, *n* (%)	206 (72)	79 (28)	109 (49)	111 (51)
Age, mean ± SD	70 ± 10	66 ± 12	71 ± 11	68 ± 14
Race, *n* (%)				
European American	191 (73)	71 (27)	94 (47)	104 (53)
African American	5 (83)	1 (17)	10 (83)	2 (17)
Gender, *n* (%)				
Male	90 (76)	28 (24)	79 (49)	83 (51)
Female	116 (69)	51 (31)	30 (53)	27 (47)
SHS exposure, *n* (%)	8	—	6	—
Histology, *n* (%)				
Adenocarcinoma	161 (69)	74 (31)		
Squamous cell carcinoma	44 (90)	5 (10)		
Stage, *n* (%)				
1A	41 (69)	18 (31)		
1B	31 (72)	12 (28)		
2A	39 (76)	12 (24)		
2B	11 (73)	4 (27)		
3A	17 (74)	6 (26)		
3B	9 (75)	3 (25)		
4	51 (69)	23 (31)		

### Liquid chromatography and tandem mass spectrometry conditions

Metabolite quantitation was performed by multiple-reaction monitoring transition an Acquity UPLC/Xevo TQ-S micro system (Waters Corp.) following an optimized quantitative protocol using synthetic heavy labeled CR (CR-^13^C,^15^N_2_, ISTD, ≥99.0%) and NANA (NANA-^13^C_3_, ISTD, ≥99.0%) internal standards. Previous publications described a detailed methodology (24, 27).

### Urine metabolite quantification

The concentrations of CR and NANA were determined by applying UPLC–MS/MS. The monophasic extraction technique was applied for urine sample preparation. Urine was diluted at 1:6 with ice-cold acetonitrile:H_2_O:methanol (65:30:5, v/v/v) buffer-1 containing 3 μmol/L CR-^13^C,^15^N_2_, and NANA-^13^C_3_ for the HILIC mode. The samples were vortexed and centrifuged at 15,000 *g* for 15 minutes. Using ice-cold acetonitrile:H_2_O (75:25, v/v) buffer-2, supernatants were taken and diluted at a 1:2 volume. Data were acquired in the positive ESI mode and processed using the Target-Lynx software (Waters Corp.). Areas under chromatographic peak for each metabolite were extracted and normalized to those for corresponding CR and NANA heavy atom-labeled internal standards. Concentrations of CR and NANA in the urine samples were calculated using calibration curves built from authentic standard solutions. Urinary metabolite levels were subjected to correlation analysis with clinical variables after normalizing to urinary creatinine, as assessed by the Jaffe method (28).

### Statistical analyses

Participants were divided into low and high groups according to the median levels of CR and NANA. To assess the diagnostic value of the CR and NANA metabolites in the study participants, receiver operating characteristic (ROC) analyses were performed using the pROC package in R language (29). The diagnostic ability of the prediction model was evaluated by calculating the AUC. Ninety-five confidence intervals (CI) are shown. The ROC curve in population controls versus lung cancer cases used for evaluation was set by generalized linear model analysis adjusted for age, race, and sex. Nonparametric statistical analysis was performed to compare the medians across nonsmokers and smokers by the Kruskal–Wallis one-way analysis of variance using the ggplot2. Box plots were used to display a summary of a set of data containing the minimum, first quartile, median, third quartile, and maximum, whereas violin plots were used to show the distribution of data. Considering the data did not follow a normal distribution and had significant outliers, the median values were regarded as a measure of central tendency. The R packages “survival” and “survminer” were used for the Kaplan–Meier (K–M) survival analysis (Log-rank test). The Cox proportional hazard regression model was applied to perform a multivariate Cox proportional hazard analysis of all factors in the study participants. The models were adjusted for variables age, race, and sex. A factor with a hazard ratio (HR) >1 was considered a risk. All statistical analyses were performed by R software (version 4.0.5). All *P* values less than or equal to 0.05 were considered statistically significant.

### Data availability

The deidentified data generated in this study are available within the article and its supplementary data files. Raw data are generated at the National Cancer Institute, National Institutes of Health (United States) and can be obtained upon reasonable request from the corresponding author.

## Results

### Elevated urinary CR and NANA levels in nonsmoker and smoker cases as compared with the population control group

**Figure 1.**
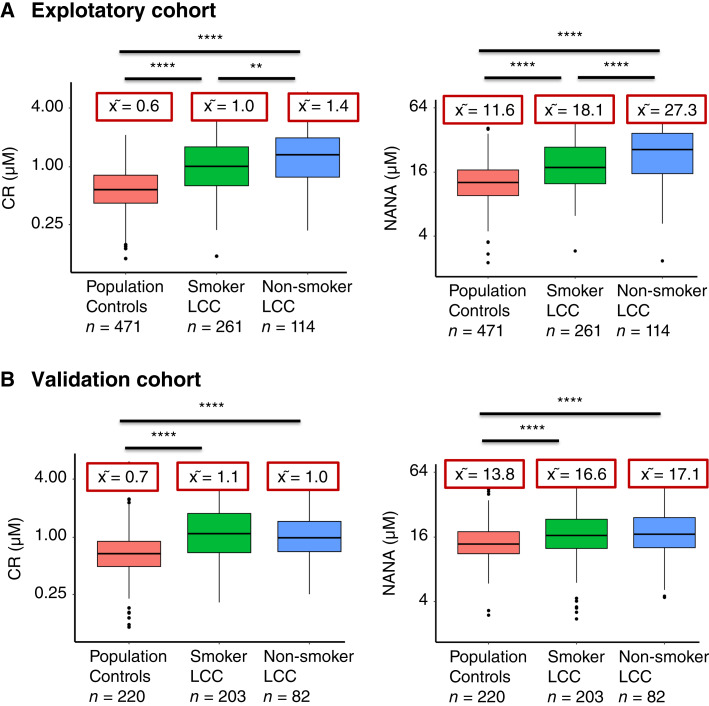
Box plots showing the distribution of CR and NANA urinary metabolite levels in the (**A**) exploratory cohort and (**B**) validation cohort were quantitatively measured by UPLC–MS/MS in the study participants. Kruskal–Wallis ANOVA; posthoc multiple comparisons, ****P < 0.0001; **P < 0.01. LCC, lung cancer cases; x͂ = median.

**Figure 2.**
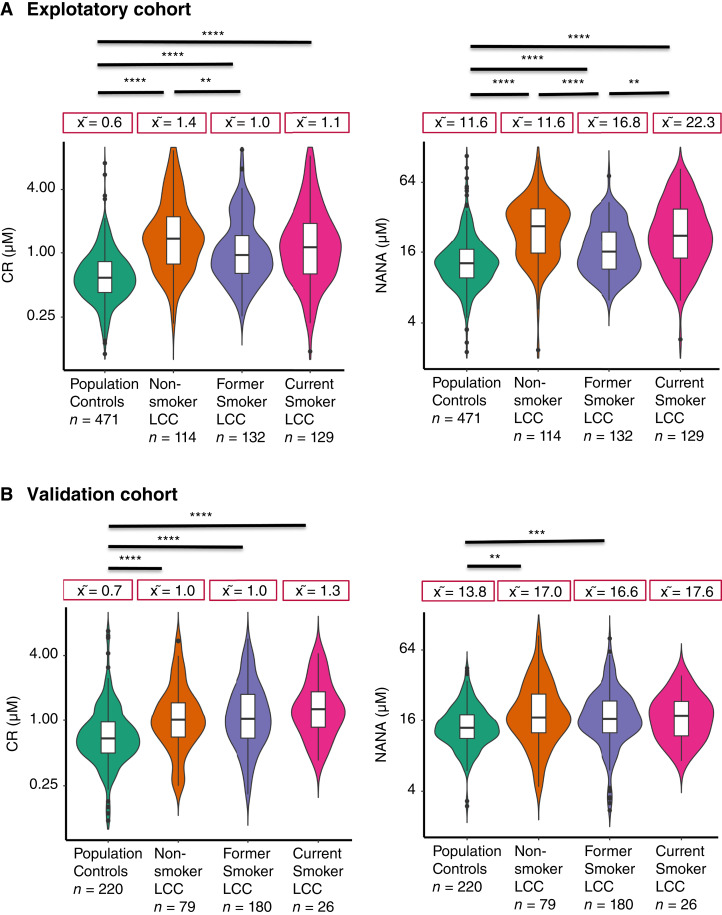
Distribution of CR and NANA metabolite levels in former and current smoker cases. Violin plots were used to depict the summary statistics and the density of each variable in different groups within the (**A**) exploratory cohort and (**B**) validation cohort. ****P < 0.0001; ***P < 0.001; **P < 0.01; ns, not significant; x͂ = median.

Participants were categorized into two groups, i.e., people with active tobacco use and people with a history of active tobacco exposure (current and former smokers) and nonsmokers. The distributions of median CR and NANA levels were compared between cases and population controls depending upon their smoking status in both exploratory and validation cohorts. In the exploratory cohort (Fig. 1A), the respective median CR and NANA levels in cases were significantly higher in nonsmokers (1.4 and 27.3 μmol/L, respectively) than smokers (1.0 and 18.1 μmol/L, respectively) and population controls (0.6 and 11.6 μmol/L, respectively). For the validation cohort (Fig. 1B), respective median CR and NANA levels in cases were significantly higher in both nonsmokers (1.0 and 17.1 μmol/L, respectively) and smokers (1.1 and 16.6 μmol/L, respectively) compared with population controls (0.7 and 13.8 μmol/L, respectively). As no significant differences were observed in CR and NANA median values based on smoking status among the population controls, they were merged. Further, no significant difference was observed in CR levels between former (1.0 μmol/L) and current smoker (1.1 μmol/L) cases in either the exploratory or validation cohorts (former smoker: 1.0 μmol/L and current smoker: 1.3 μmol/L), whereas NANA levels were significantly increased in the current smoker (22.3 μmol/L) compared with former smoker (16.8 μmol/L) cases in the exploratory cohort, and no statistically significant difference was found between current smoker (17.6 μmol/L) compared with former smoker (16.6 μmol/L) cases in the validation cohort (Fig. 2). We then focused on metabolite levels in cases with secondhand smoking exposure. As shown in Supplementary Fig. S1A, the respective median CR and NANA levels were significantly higher for those without childhood parental smoking (CPS) exposure (1.3 and 34.9 μmol/L, respectively) and with CPS exposure (1.4 and 25.7 μmol/L, respectively) nonsmoker cases compared with population controls (0.6 and 11.5 μmol/L, respectively), whereas as shown in Supplementary Fig. S1B, the respective median CR and NANA levels were significantly higher for those without SHS exposure (1.4 and 31.5 μmol/L, respectively) and with SHS exposure (1.4 and 26.6 μmol/L, respectively) nonsmoker cases compared with population controls (0.6 and 11.5 μmol/L, respectively).

### Comparison of urinary CR and NANA metabolite levels by histology and stage

Next, we examined the distributions of CR and NANA in NSCLC cases with adenocarcinomas only compared with population controls and identified significant differences in the levels of these metabolites according to smoking status within this histological subtype (Supplementary Fig. S2A). Specifically, respective median CR and NANA levels were significantly higher in nonsmoker cases (1.4 and 27.3 μmol/L, respectively) than cases who smoked (1.0 and 17.7 μmol/L, respectively), which were both higher than population controls (0.6 and 11.6 μmol/L, respectively). By contrast, for the validation cohort, the respective median CR and NANA levels were higher in both nonsmoker cases (1.0 and 17.0 μmol/L, respectively) and cases who smoked (1.0 and 16.3 μmol/L, respectively) than population controls (0.7 and 13.8 μmol/L, respectively). Cases were further stratified by early stage (I and II) and late stage (III and IV) to evaluate the CR and NANA levels among early-stage cases, late-stage cases, and population controls grouped by their smoking status (Supplementary Figs. S3 and S4). The respective median CR and NANA levels for the exploratory cohort were significantly higher in nonsmokers (1.5 and 26.9 μmol/L, respectively) than in smokers in early-stage cases (0.9 and 17.5 μmol/L, respectively) and population controls (0.6 and 11.6 μmol/L, respectively). The respective median CR and NANA levels for the validation cohort were higher in nonsmokers (0.9 and 16.3 μmol/L, respectively) and smokers in early-stage cases (0.9 and 16.3 μmol/L, respectively) than the population controls (0.7 and 13.8 μmol/L, respectively). The median CR levels were elevated in both late-stage nonsmoker cases (1.4 μmol/L) and cases who smoked (1.6 μmol/L) compared with population controls (0.6 μmol/L). The median NANA levels were significantly higher in nonsmokers (31.3 μmol/L) than smokers in late-stage cases (22.2 μmol/L) and population controls (11.6 μmol/L). The respective median CR and NANA levels for the validation cohort were higher in nonsmokers (1.5 and 19.7 μmol/L, respectively) and smokers in late-stage cases (1.3 and 16.8 μmol/L) than the population controls (0.7 and 13.8 μmol/L).

### Comparison of urinary CR and NANA metabolite levels by race and sex

The CR and NANA levels for the exploratory cohort were evaluated in the African‐American (AA) and European‐American (EA) populations overall, irrespective of smoking status as very few nonsmoker African-American study participants were available (Supplementary Fig. S5A). The median CR levels were significantly higher in both AA cases (1.1 μmol/L) and EA cases (1.1 μmol/L) than population controls (AA; 0.6 μmol/L and EA; 0.6 μmol/L), whereas the median NANA levels were significantly higher in EA cases (22.1 μmol/L) than AA cases (18.0 μmol/L) and population controls (AA; 11.2 μmol/L and EA; 11.8 μmol/L). The median CR and NANA levels for the validation cohort (Supplementary Fig. S5B) were significantly higher in both AA cases (1.4 and 20.4 μmol/L, respectively) and EA cases (1.0 and 16.9 μmol/L, respectively) than population controls (AA; 0.6 μmol/L, EA; 0.7 μmol/L and AA; 12.9 μmol/L, EA; 14.0 μmol/L). Next, the distributions of CR and NANA for the exploratory cohort (Supplementary Fig. S6A) in nonsmokers revealed that the median CR levels were significantly higher in both male (1.3 μmol/L) and female (1.4 μmol/L) cases than population controls (male; 0.6 μmol/L; female; 0.6 μmol/L), whereas the median NANA levels were significantly higher in female (28.2 μmol/L) than male (22.0 μmol/L) cases and population controls (male; 9.5 μmol/; female; 12.8 μmol/L). The distributions of CR and NANA for the validation cohort (Supplementary Fig. S6B) revealed that the median CR levels were significantly higher in both male (1.0 μmol/L) and female (1.0 μmol/L) cases than population controls (male; 0.7 μmol/L; female; 0.8 μmol/L), whereas the median NANA levels were significantly higher in male (16.5 μmol/L) than population controls (13.5 μmol/L). The median NANA levels were significantly higher in female (18.3 μmol/L) than male (16.5 μmol/L) cases.

### Survival and ROC analysis

**Figure 3.**
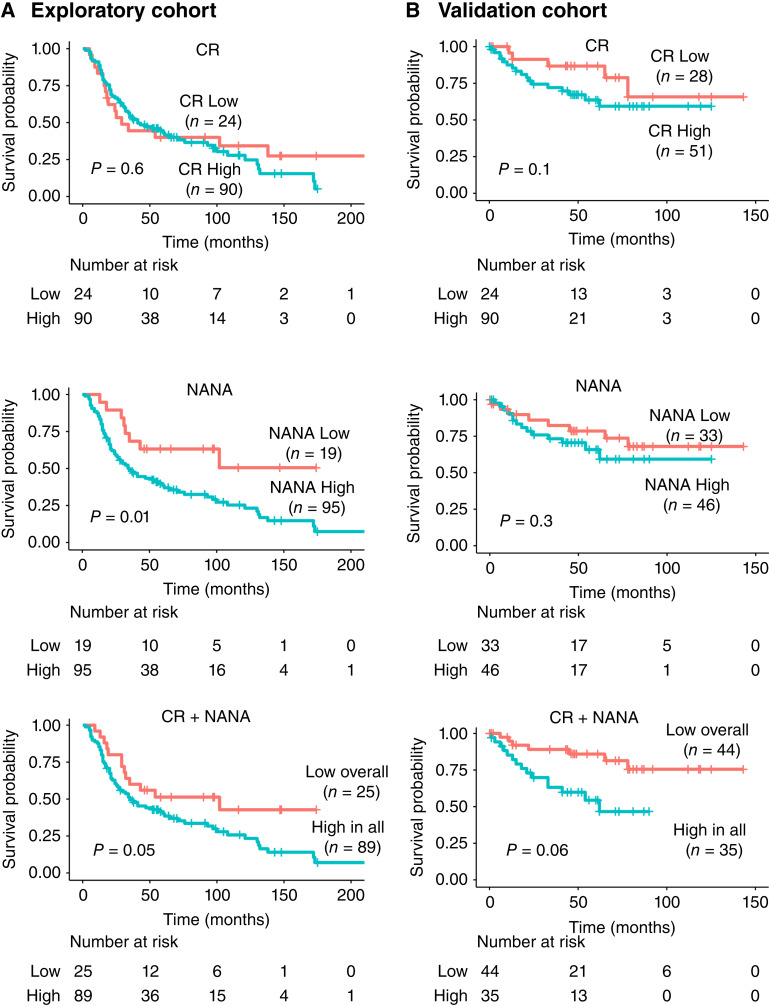
K–M plots of the overall survival of lung cancer cases stratified by the median cutoff value of CR and NANA for nonsmokers in the (**A**) exploratory cohort and (**B**) validation cohort.

**Figure 4.**
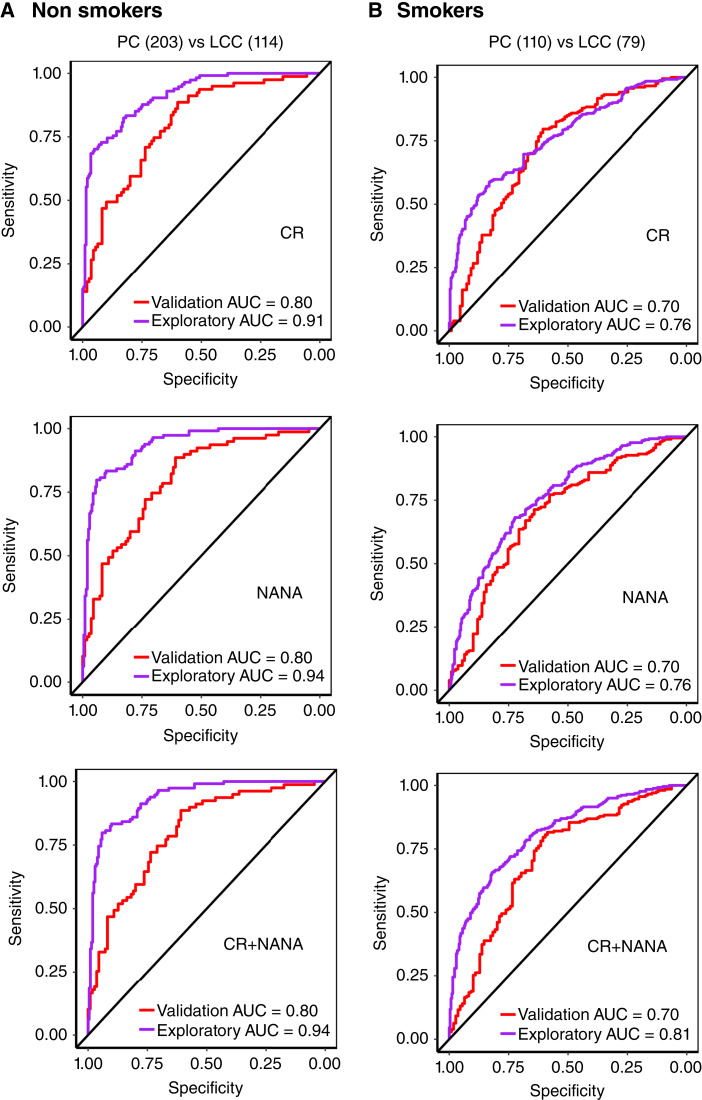
ROC curves represent the accuracy of metabolite CR, NANA, and the combination with AUC for smoking status in both cohorts: (**A**) nonsmokers and (**B**) smokers. PC, population controls.

Metabolite levels were dichotomized into high and low categorical variables based on median cutoff values each for the exploratory cohort (CR: 0.7 μmol/L; NANA: 14.6 μmol/L) and the validation cohort (CR: 0.8 μmol/L; NANA: 15.4 μmol/L). Log-rank K–M plot analysis for the exploratory cohort showed that the higher levels of NANA were significantly associated with poor survival in nonsmoker cases, and for CR, it was not associated with survival (Fig. 3A). Furthermore, both biomarkers are significantly associated with survival in cases who smoked (Supplementary Fig. S7A). Additionally, when both metabolites were combined, patients with high CR and NANA levels had significantly worse survival outcomes than those with either low CR or low NANA levels in nonsmoker cases (Fig. 3A), as well as cases who smoked (Supplementary Fig. S7A). Log-rank K–M plot analysis for the validation cohort showed that the higher levels of CR or NANA were not associated with poor survival in nonsmoker cases (Fig. 3B), but significantly associated with cases who smoked (Supplementary Fig. S7B). Further, when both metabolites were combined, patients with high CR and NANA had significantly worse survival outcomes than those with either low CR or NANA in nonsmoker cases (Fig. 3B), as well as cases who smoked (Supplementary Fig. S7B). Survival estimates for nonsmokers and those who smoked showed no significant difference when stratified by stage and histology as shown in Supplementary Fig. S8. ROC curve analysis was performed to evaluate the diagnostic accuracy for smoking status, i.e., the nonsmokers and smokers group. ROC analysis with AUC (95% CI) for nonsmokers (Fig. 4A) resulted in 0.91 (0.88–0.94) in the exploratory cohort compared with the validation cohort 0.8 (0.74–0.86) for CR metabolite and 0.94 (0.91–0.96) in the exploratory cohort compared with the validation cohort 0.8 (0.69–0.83) for NANA metabolite. The ROC analysis for combined CR and NANA levels resulted in a better prediction of 0.94 (0.91–0.96) in the exploratory cohort compared with the validation cohort of 0.8 (0.74–0.86). Combining both metabolites leads to a positive predictive value of 89% and 62%, and a negative predictive value of 88% in the exploratory and validation cohorts. The diagnostic efficiency of models in nonsmokers for lung cancer cases and population controls as well as for early and late stages is described in Supplementary Table S1. Among those who smoked (Fig. 4B), ROC analysis resulted in 0.76 (0.72–0.80) in the exploratory cohort compared with the validation cohort 0.7 (0.66–0.79) for CR metabolite and 0.76 (0.72–0.80) in the exploratory cohort compared with the validation cohort 0.7 (0.64–0.76) for NANA metabolite. The ROC analysis for combined CR and NANA levels resulted in a better prediction of 0.81 (0.77–0.85) in the exploratory cohort compared with the validation cohort of 0.7 (0.65–0.78).

### Univariate and multivariate analysis

The metabolite levels, clinical factors, and prognosis in nonsmokers were analyzed by univariate and multivariate regression analyses (Table 2). CR + NANA high-metabolite category (*P* = 0.04) and stage (*P* <0.001) were identified as significant indicators of survival in the univariate analysis for the exploratory cohort, whereas the CR + NANA high-metabolite category (*P* = 0.006), stage (*P* = 0.002), and sex (*P* = 0.05) were identified as significant indicators of survival in the univariate analysis for the validation cohort. In multivariate analysis for the exploratory cohort, cancer stage, histology, and CR + NANA high-metabolite category were found to be independent predictors of survival (*P* < 0.05). In the validation cohort, cancer stage and CR + NANA high-metabolite category were again independent predictors of survival (*P* < 0.05). Univariate and multivariate analyses of factors associated with survival in cases of smokers is shown in Supplementary Table S2. The multivariate analysis data suggest that CR and NANA may be more significantly associated with survival in nonsmokers than in smokers in both cohorts. Notably, smoking history (Supplementary Fig. S9) was not a significant factor in the Cox proportional hazard model.

**Table 2.** Univariate and multivariate regression analysis for factors associated with survival in nonsmokers from both cohorts.

Exploratory cohort		Univariable				Multivariable		
Factors	Levels	*N*	HR	95% CI	*P *value	HR	95% CI	*P *value
CR + NANA category	Low; high	114	1.79	0.99–3.24	0.041	2.74	1.40–5.38	0.001
Stage	Early stage (I and II); late stage (III and IV)	107	3.26	1.99–5.33	<0.001	3.83	2.26–6.51	<0.001
Histology	Adenocarcinoma; squamous cell carcinoma	114	1.62	0.74–3.55	0.26	3.44	1.49–7.95	0.011
Age	Years	114	0.99	0.97–1.00	0.086	0.99	0.98–1.01	0.4
Sex	Male; female	114	0.95	0.59–1.52	0.82	0.85	0.50–1.47	0.6
Race	African American; European American	114	0.84	0.26–2.67	0.77	0.39	0.12–1.33	0.2

Validation cohort		Univariable				Multivariable		
Factors	Levels	*N*	HR	95% CI	*P *value	HR	95% CI	*P *value
CR + NANA category	Low; high	79	3.37	1.35–8.39	0.006	2.92	1.13–7.50	0.022
Stage	Early stage (I and II); late stage (III and IV)	79	3.99	1.55–10.2	0.002	2.71	0.99–7.42	0.042
Histology	Adenocarcinoma; squamous cell carcinoma	79	0.88	0.12–6.59	0.90	0.79	0.08–8.04	0.8
Age	Years	79	0.98	0.95–1.02	0.33	0.99	0.95–1.04	0.7
Sex	Male; female	79	0.43	0.19–0.99	0.050	0.55	0.23–1.32	0.2
Race	African American; European American	79	1.65	0.44–6.18	0.48	1.54	0.39–6.04	0.6

## Discussion

Previously, we reported that urinary CR and NANA are diagnostic and prognostic biomarkers of lung cancer, and we validated the same in tumor tissues of patients with NSCLC (24). Our current analysis indicates that urinary CR and NANA are significantly elevated in lung cancer cases from nonsmokers and those who smoked when compared with population controls. Further, no significant association was found in CR levels in former and current smoker cases, whereas NANA levels were significantly (*P* < 0.01) increased in current smokers as compared with former smoker cases only in the exploratory cohort. The median levels of CR and NANA were found to be elevated in nonsmoker cases with and without SHS and CPS exposure compared with population controls. No significant association was found between nonsmoker cases with or without SHS and CPS exposure. The above two findings indicate that the levels of metabolites are independent of exposure to tobacco smoking.

The incidence of SHS exposure among nonsmokers in the United States decreased significantly from 87.5% to 25.2% between 1988 and 2014 (30). SHS exposure causes more than 7,300 deaths from lung cancer each year among people who do not smoke (31). A previous study showed that amino acid and lipid metabolism pathways are the most significantly affected by exposure to tobacco smoking (32). However, CR and NANA are not members of these pathways (32). A study on diagnostic and prognostic evaluation showed that CR and NANA were also significantly elevated in intrahepatic cholangiocarcinoma (ICC) when compared with hepatocellular carcinoma cases in both urine and tissue samples. High levels of CR were associated with a poorer prognosis in ICC (33), indicating that CR levels are not lung cancer specific.

Over the last three decades, lung adenocarcinoma has steadily surpassed squamous cell carcinoma as the most prevalent histological form, particularly among nonsmokers (34). Adenocarcinomas account for approximately 50% to 60% of lung malignancies identified in adults who do not smoke. In the exploratory cohort, significantly elevated CR and NANA levels were found in nonsmoker adenocarcinoma cases compared with adenocarcinoma cases and population controls who smoked. Early diagnosis has a large impact on lung cancer outcomes, with 5-year relative survival increasing from 6% for metastatic-stage disease to 33% for regional stage and 60% for localized-stage disease (1). The survival associated with early-stage NSCLC is less than optimal with a 5-year overall survival and was lowest for patients with stage IV NSCLC. Most nonsmokers do not show early signs and symptoms of lung cancer and most often, when early-stage lung cancer is found in nonsmokers it is due to incidental findings. In this study, the estimated CR and NANA levels were significantly (*P* < 0.05) higher in early-stage nonsmoker cases than the population controls in the exploratory cohort, which indicates that these metabolites can be used as a diagnostic biomarker to predict the development of early-stage lung cancer in nonsmoker cases.

Lung cancer is the leading cause of cancer death among women in the United States (1). The incidence rate in women who do not smoke is higher (ranging from 14.4 to 20.8 per 100,000 person-years) than men who do not smoke (ranging from 4.8 to 13.7 per 100,000 person-years; ref. 35). Diamantopoulou and colleagues reported higher NANA levels in the serum of women with endometrial cancer (36). Increased levels of NANA metabolite were found in nonsmoking females as compared with the male lung cancer cases in the exploratory as well as validation cohorts. Lung cancer is the most common cause of cancer death among AA men and the second-leading cause in AA women with an estimated diagnosis of 25,690 AA people in 2022. The survival rate for lung cancer is slightly lower in the AA population than in the EA population overall, comparable with a previous study (37). In our previous study, we showed that CR and NANA were associated with lung cancer risk more significantly in EA (38). This study showed significantly (*P* < 0.05) increased levels of NANA metabolite in EA cases compared with the population controls in the exploratory cohort, which further confirms clinical utility for disease screening or diagnosis.

Urine is noninvasive, abundant in volume, and requires minimal preparation for analysis. Our previous prospective study reported that CR and NANA had a positive association with increasing tumor size (38). These biomarkers are also detectable in other liquid biopsies such as serum and plasma. CR is a tumor-derived metabolite detectable in serum and plasma biospecimens, adding to prior investigations of CR as a urine biomarker of risk and prognosis in other cancer types (24, 33, 38, 39). Alternatively, NANA is the most abundant sialic acid in human serum. In the absence of urine samples, these biomarkers can be identified in serum and plasma.

These results indicate that urinary levels of CR and NANA either alone or in combination might be useful for the stratification of patients with early-stage NSCLC having a worse prognosis after surgery and could be a potential prognostic biomarker. The high levels of urinary metabolites could lead nonsmoker individuals to take LDCT screening even if they do not have current eligibility. High levels of urinary CR and NANA metabolites category were associated with poor lung cancer survival for both nonsmokers and those who smoked in both cohorts. ROC analysis in the nonsmokers had higher AUC with both the urinary metabolites alone and in combination than the smoker group in both cohorts and for the nonsmoker population, CR + NANA metabolite category factor and stage were independent predictors of survival in multivariate analyses for both cohorts. Further, Cox proportional analysis in lung cancer cases for both cohorts showed that smoking was not a significant predictor of survival. Wu and colleagues' risk assessment tool comprising two serum biomarkers (alpha-fetoprotein and carcinoembryonic antigen) reported an AUC of 0.806 for nonsmokers but it seems to be without validation data, hence it is likely to be overstated (40). In another study, a serum combinational biomarker for early-stage lung cancer could distinguish nonsmoking female cases from healthy controls. The biomarkers performed very well with high specificity and sensitivity but had only female participants and very few nonsmoker cases (41). Geng and colleagues' study reported upregulation of *miR-155* in plasma for early-stage (I and II) nonsmoker cases (*n* = 37) compared with healthy controls. Combinations of miRNAs as screening tools have the potential to be more precise than single miRNAs used in the study and have a limitation of a relatively small sample size (42).

The present study has some limitations. For example, the study reported here was in a retrospective setting. To determine the clinical utility of diagnostic and prognostic biomarkers, prospective trials are needed for nonsmokers. Our previous metabolic efforts with a prospective cohort study comprising mostly smokers confirmed CR as predictive of the lung cancer risk before radiographically visible cancers and NANA displaying borderline significance. Also, we could not validate biomarker levels in lung cancer among those who do not smoke in the Asian population, and we do not know if it will have the same predictive ability for other racial/ethnic groups.

In the present study, we did not test any metabolic pathway association for the increase of these metabolites in lung cancer cases. In a previous metabolic study, we showed that CR is a companion-diagnostic biomarker and elevated CR levels indicate a diversion of mitochondrial urea cycle metabolites to promote nucleotide synthesis, leading to urea cycle dysregulation and arginine auxotrophy in multiple types of human cancers and a biomarker of response in urea cycle dysregulation–targeted therapy in liver cancer (43). This highlights the potential of CR not only as a prognosis biomarker but also as a companion biomarker of arginine-targeted therapies in precision medicine strategies to improve cancer patient survival. NANA is the most common sialic acid derivative in humans (44). The synthesis of sialylated glycans utilizes Golgi-resident, membrane-bound sialyltransferase enzymes. Several malignancies, including lung, breast, ovarian, pancreatic, and prostate cancer, are known to upregulate these enzymes in 40% to 60% of tumor cell surfaces and are more commonly observed in tumor tissues compared with normal tissues (44). Hypersialylation accelerates cancer progression and leads to poor prognosis. The increased sialic acids in tumor cells cause the special metabolic flux and aberrant expression of sialyltransferases (44).

Overall, the results of this study indicate that high levels of CR and NANA in the urine could be potential diagnostic and prognostic biomarkers of NSCLC, regardless of smoking. This liquid biopsy would be an inexpensive, noninvasive, and high-throughput method to facilitate cancer detection, improve lung cancer risk assessment, define eligibility for LDCT screening, and indicate the need for therapy in early-stage cases to improve lung cancer survival outcomes.

## Supplementary Material

Supplementary Figure S1 Distribution of CR and NANA metabolite levels in non-smoker cases without and with (A) Childhood parental smoking exposure and (B) Secondhand smoking exposure. **** p<0.0001; NS, non-smokers; CPS, Childhood parental smoking exposure; SHS, Secondhand smoking exposure, LCC, lung cancer cases; x͂ = median

Supplementary Figure S2 Distribution of CR and NANA metabolite levels in (A) Exploratory cohort and (B) Validation cohort adenocarcinoma cases. ****p<0.0001; LUAD, lung adenocarcinoma; x͂ = median

Supplementary Figure S3 Distribution of CR and NANA metabolite levels in early-stage (I & II) cases. ****, p < 0.0001, ***, p < 0.001; ES, early-stage (I & II); x͂ = median

Supplementary Figure S4 Distribution of CR and NANA metabolite levels in late-stage (III & IV) cases. **** p<0.0001, *** p<0.001; LS, late-stage (III & IV); x͂ = median

Supplementary Figure S5 Distribution of CR and NANA metabolite levels in AA and EA participants. **** p<0.0001* p<0.05; AA, African American; EA, European American; LCC, lung cancer cases; x͂ = median

Supplementary Figure S6 Distribution of CR and NANA metabolite levels in (A) Exploratory cohort and (B) Validation cohort in male and female participants. **** p<0.0001; * p<0.05; LCC, lung cancer cases; NS, Non-smokers; x͂ = median

Supplementary Figure S7 K-M plots of overall survival of lung cancer cases stratified by the median cutoff value of CR and NANA for smokers in (A) Exploratory cohort and (B) Validation cohort.

Supplementary Figure S8 Survival analysis for Non-smokers and Smokers by stage and histology. K-M plot for (A) Early-stage (I &II) Lung cancer cases; Late-stage (III & IV) Lung cancer cases and (B) LUAD cases; LUSC cases. NS, Non-smokers; SK, Smokers; LUAD, Lung adenocarcinoma; LUSC, Lung squamous cell carcinoma

Supplementary Figure S9 Cox proportional analysis for factors associated with survival

Supplementary Table S1 The diagnostic efficiency of models in non-smoking exploratory and validation cohorts. LCC, Lung cancer cases; PC, Population controls; AUC, area under the curve; CI, confidence interval; SN, Sensitivity; SP, Specificity; NPV, negative predictive values; PPV, positive predictive values; CR, Creatine riboside; NANA- N-acetyl neuraminic acid.

Supplementary Table S2 Univariate and multivariate regression analysis for clinical factors, metabolite levels and prognosis in smokers.
