# Supplementary figures and images for: Urinary Metabolite Diagnostic and Prognostic Liquid Biopsy Biomarkers of Lung Cancer in Nonsmokers and Tobacco Smokers
Source: Clin Cancer Res. 2024 Jun 5;30(16):3592–602. doi: 10.1158/1078-0432.CCR-24-0637 (PMC11325153; doi:10.1158/1078-0432.CCR-24-0637)

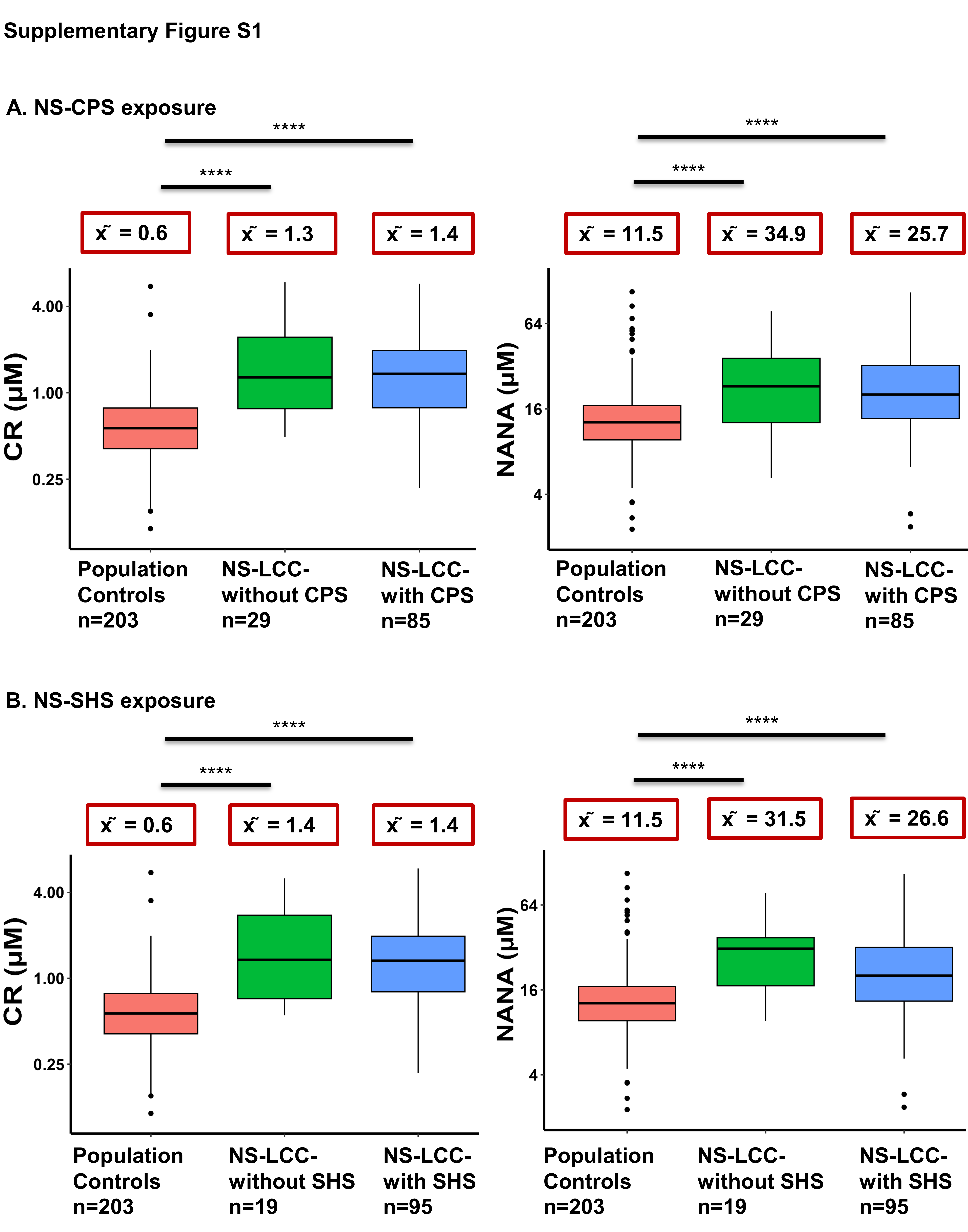

Supplement: Supplementary Figure S1 — Distribution of CR and NANA metabolite levels in non-smoker cases without and with (A) Childhood parental smoking exposure and (B) Secondhand smoking exposure. **** p<0.0001; NS, non-smokers; CPS, Childhood parental smoking exposure; SHS, Secondhand smoking exposure, LCC, lung cancer cases; x͂ = median [file ccr-24-0637_supplementary_figure_s1_suppfs1.png]

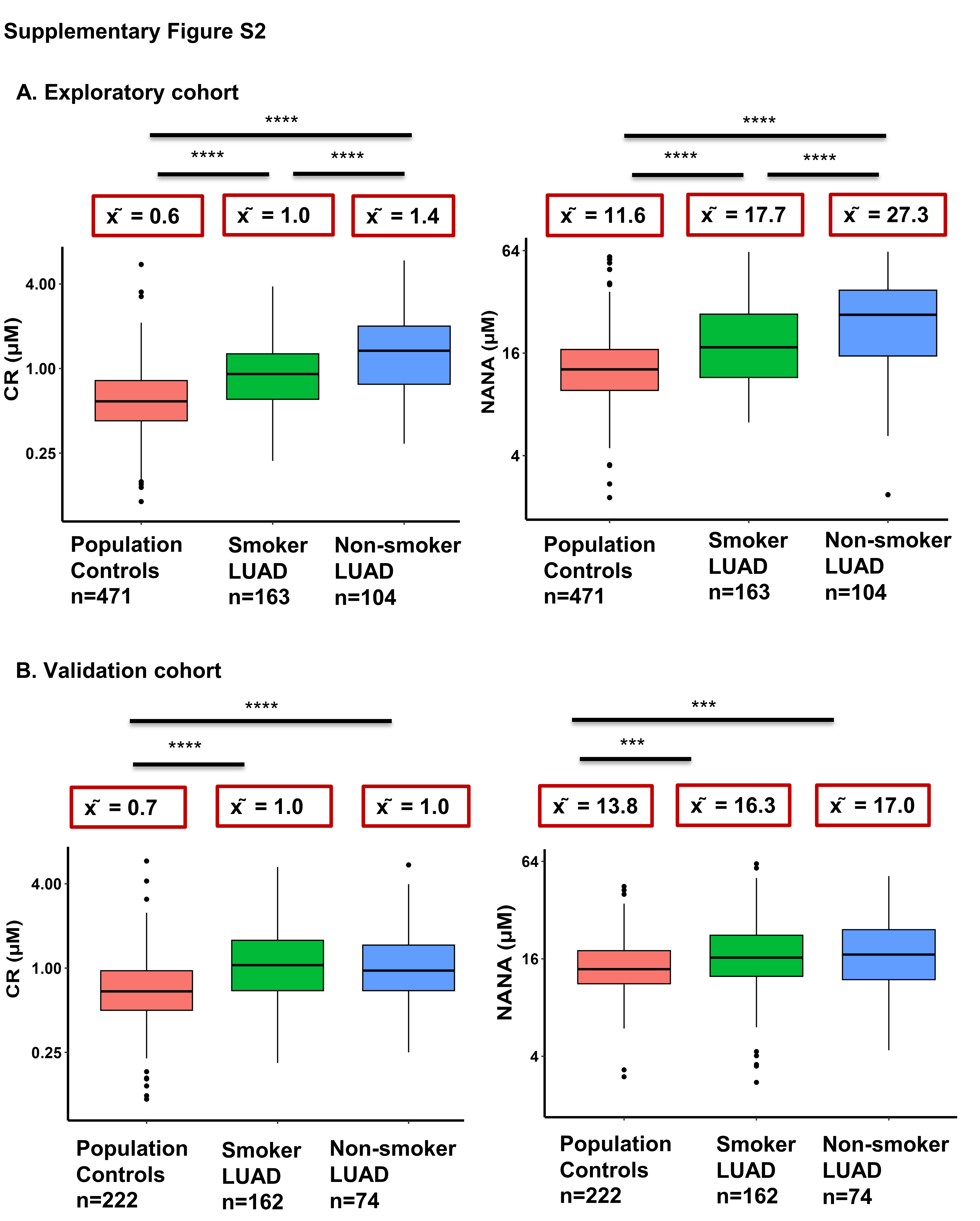

Supplement: Supplementary Figure S2 — Distribution of CR and NANA metabolite levels in (A) Exploratory cohort and (B) Validation cohort adenocarcinoma cases. ****p<0.0001; LUAD, lung adenocarcinoma; x͂ = median [file ccr-24-0637_supplementary_figure_s2_suppfs2.png]

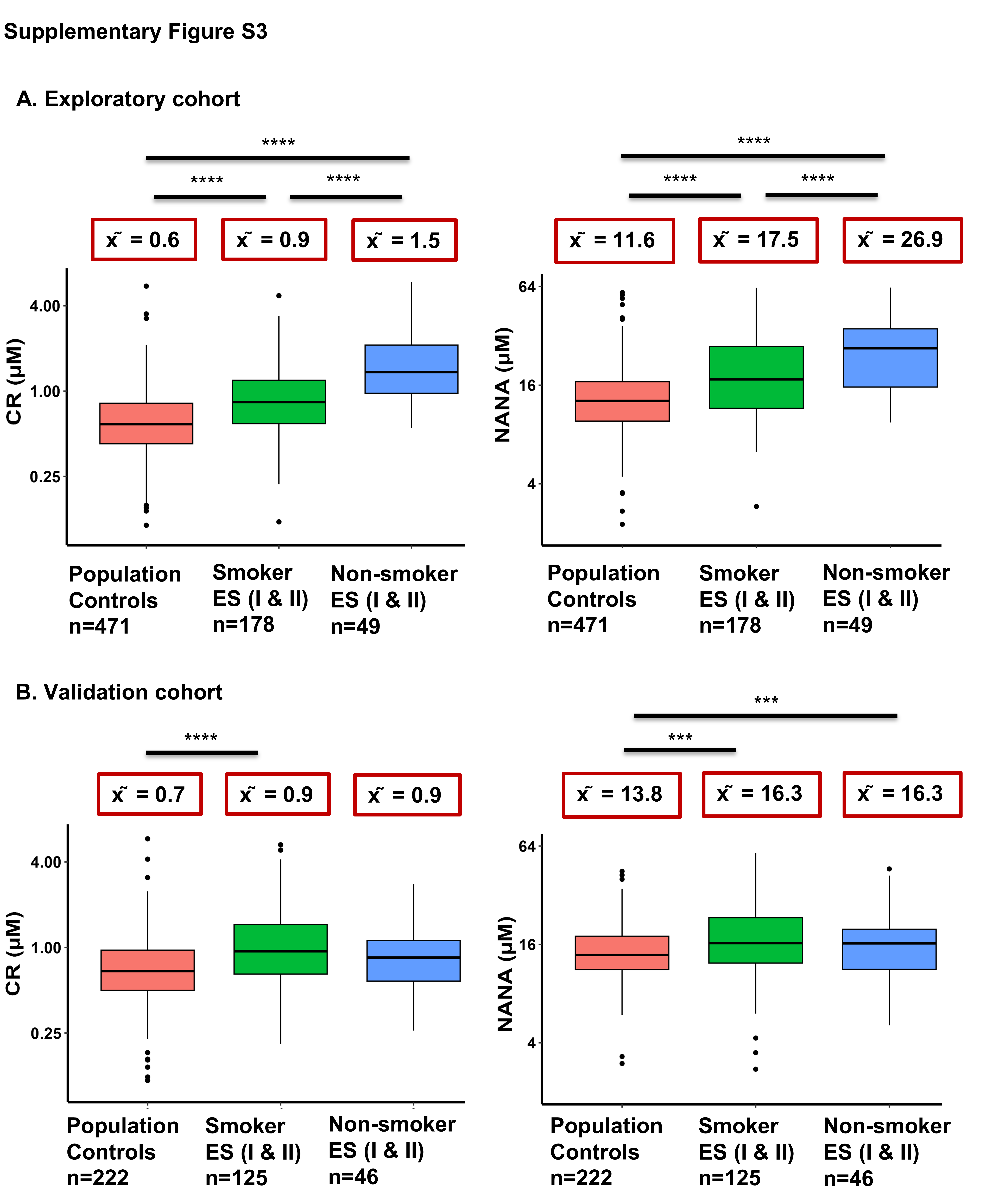

Supplement: Supplementary Figure S3 — Distribution of CR and NANA metabolite levels in early-stage (I & II) cases. ****, p < 0.0001, ***, p < 0.001; ES, early-stage (I & II); x͂ = median [file ccr-24-0637_supplementary_figure_s3_suppfs3.png]

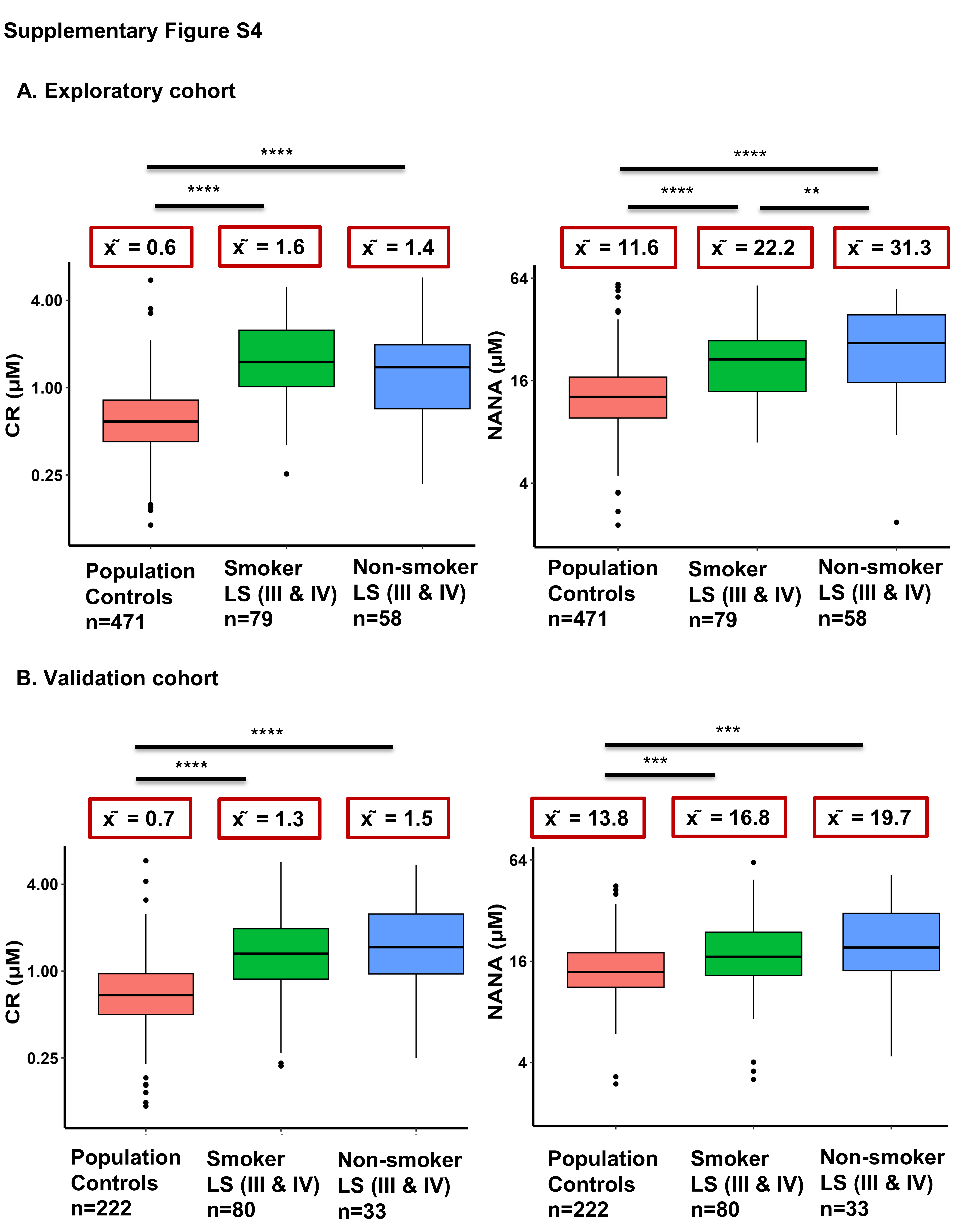

Supplement: Supplementary Figure S4 — Distribution of CR and NANA metabolite levels in late-stage (III & IV) cases. **** p<0.0001, *** p<0.001; LS, late-stage (III & IV); x͂ = median [file ccr-24-0637_supplementary_figure_s4_suppfs4.png]

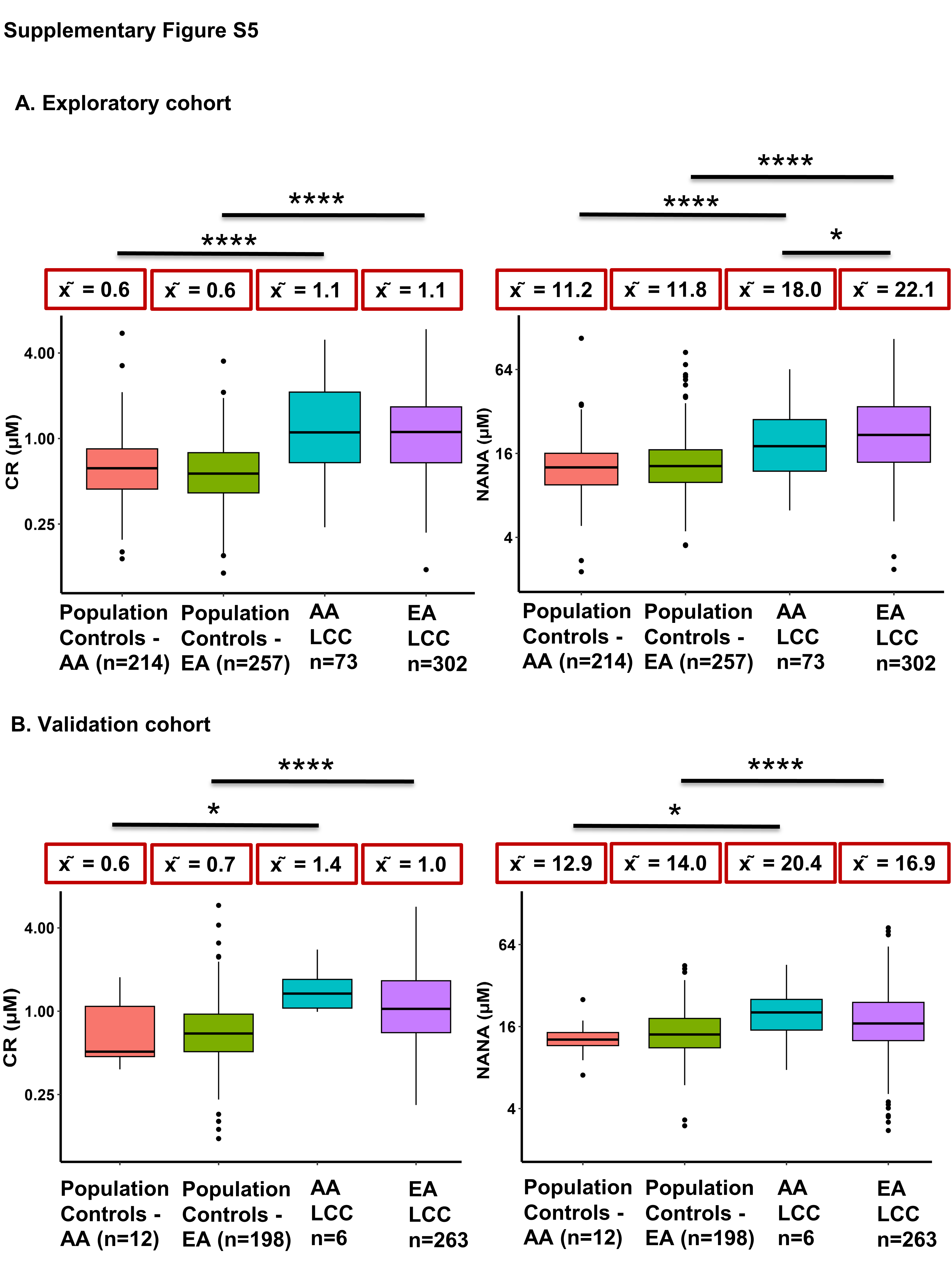

Supplement: Supplementary Figure S5 — Distribution of CR and NANA metabolite levels in AA and EA participants. **** p<0.0001* p<0.05; AA, African American; EA, European American; LCC, lung cancer cases; x͂ = median [file ccr-24-0637_supplementary_figure_s5_suppfs5.png]

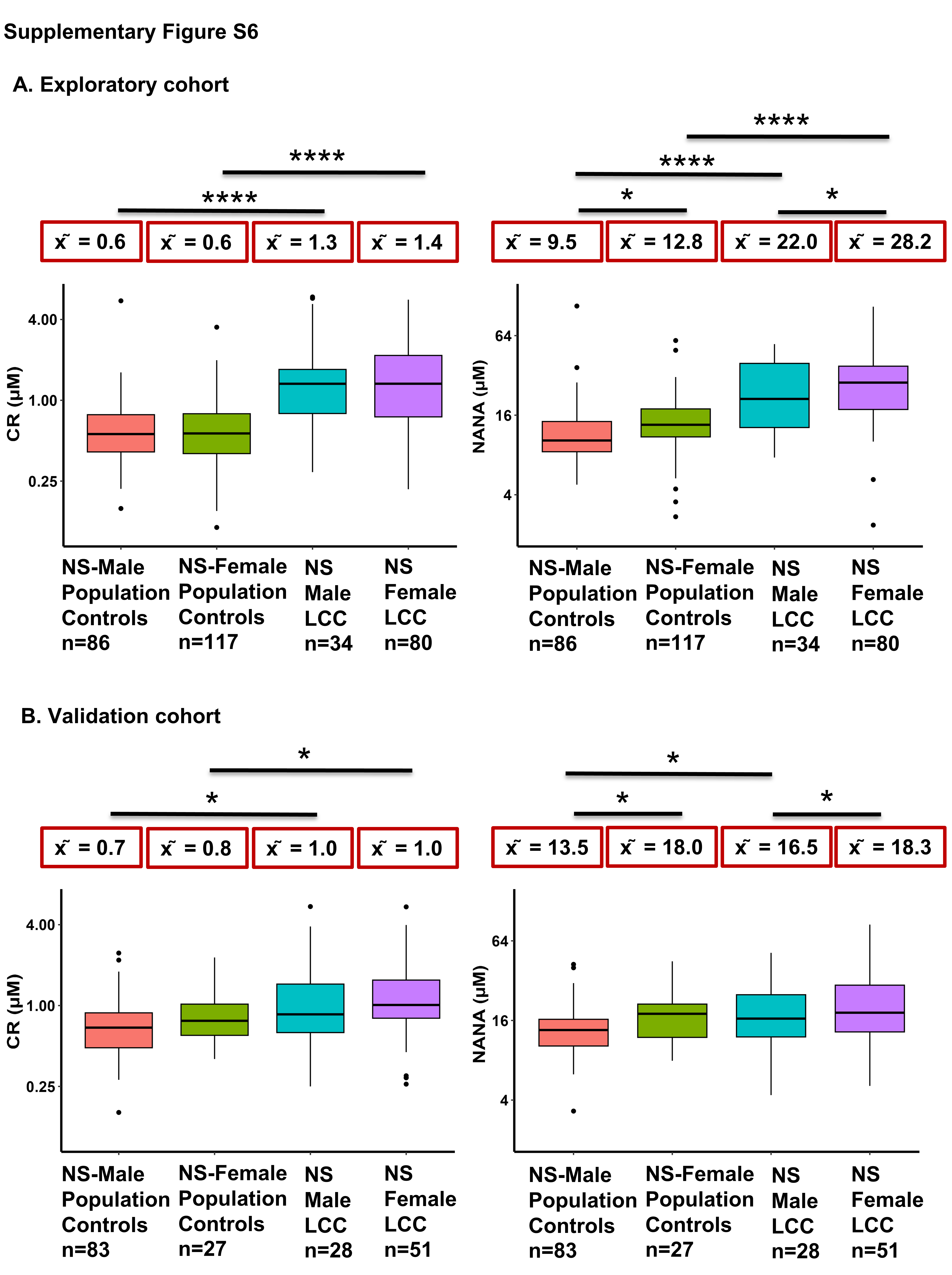

Supplement: Supplementary Figure S6 — Distribution of CR and NANA metabolite levels in (A) Exploratory cohort and (B) Validation cohort in male and female participants. **** p<0.0001; * p<0.05; LCC, lung cancer cases; NS, Non-smokers; x͂ = median [file ccr-24-0637_supplementary_figure_s6_suppfs6.png]

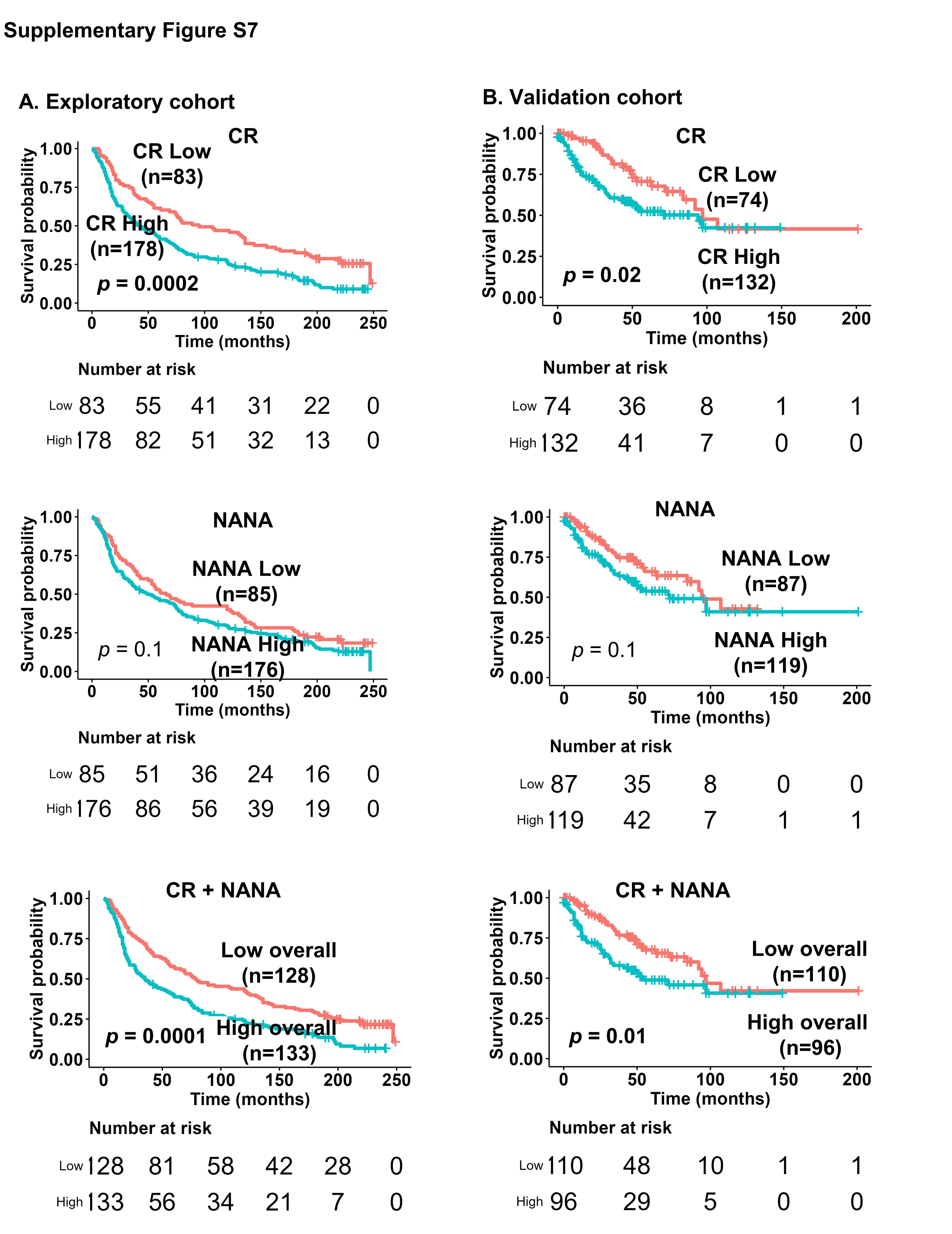

Supplement: Supplementary Figure S7 — K-M plots of overall survival of lung cancer cases stratified by the median cutoff value of CR and NANA for smokers in (A) Exploratory cohort and (B) Validation cohort. [file ccr-24-0637_supplementary_figure_s7_suppfs7.png]

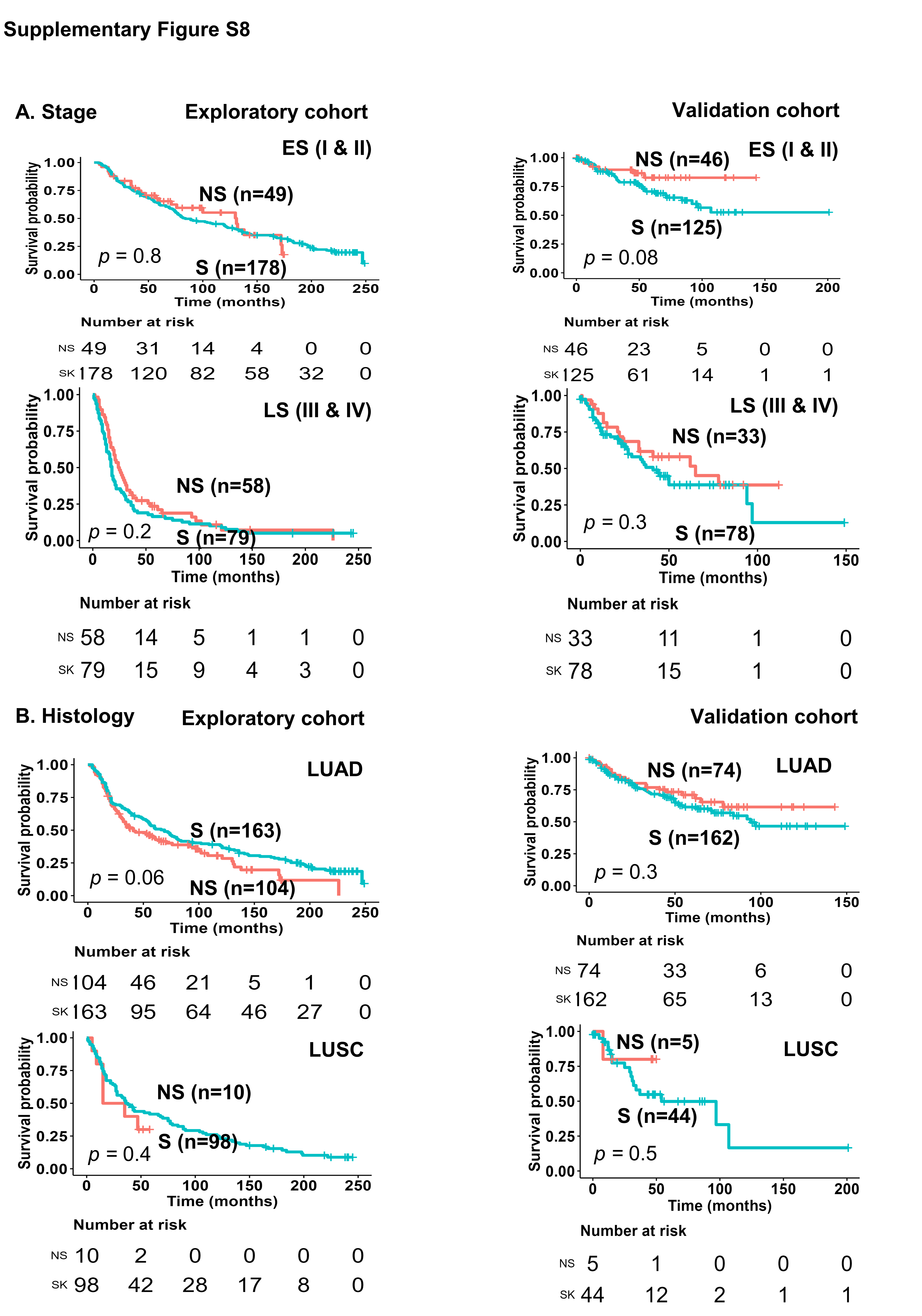

Supplement: Supplementary Figure S8 — Survival analysis for Non-smokers and Smokers by stage and histology. K-M plot for (A) Early-stage (I &II) Lung cancer cases; Late-stage (III & IV) Lung cancer cases and (B) LUAD cases; LUSC cases. NS, Non-smokers; SK, Smokers; LUAD, Lung adenocarcinoma; LUSC, Lung squamous cell carcinoma [file ccr-24-0637_supplementary_figure_s8_suppfs8.png]

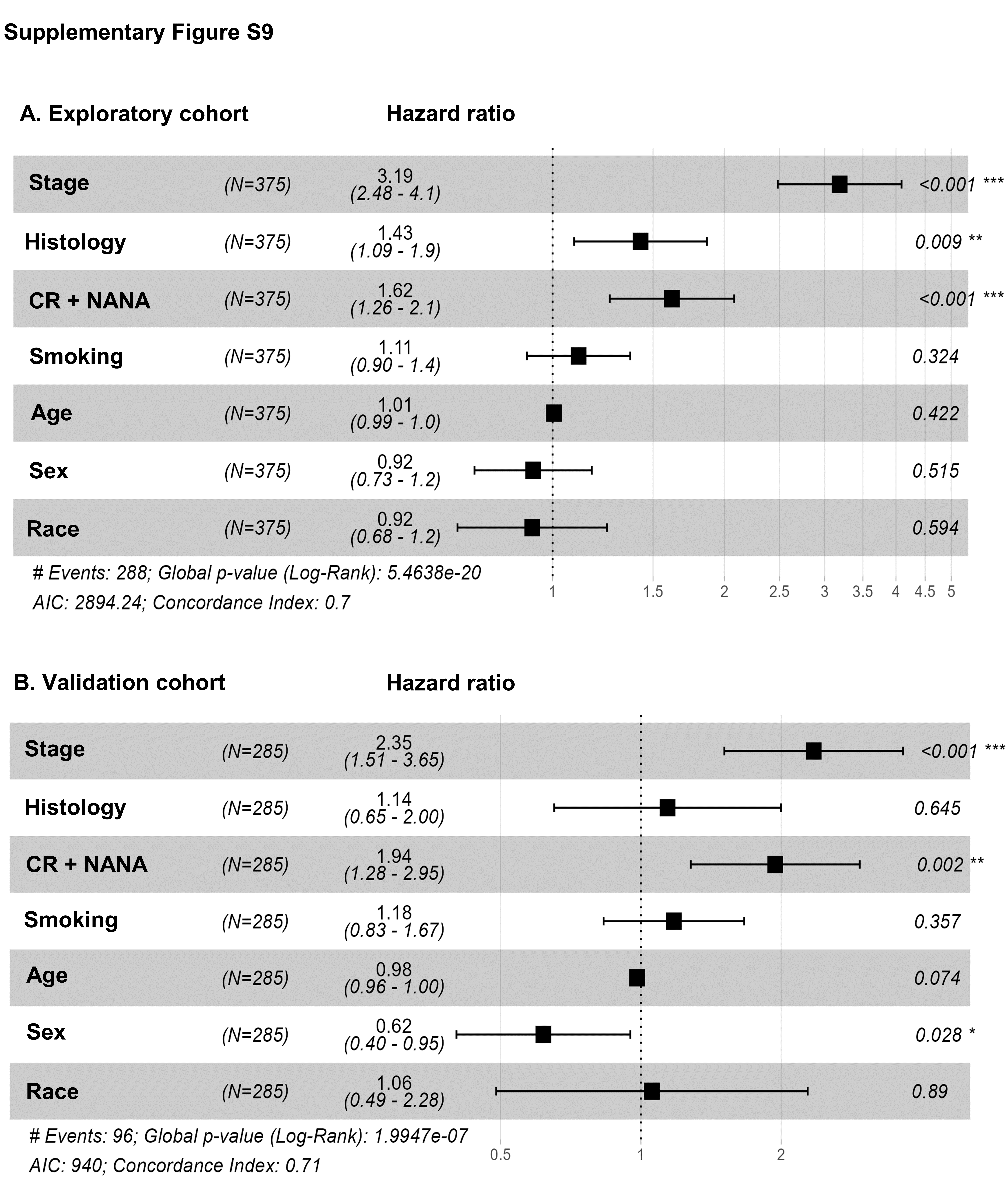

Supplement: Supplementary Figure S9 — Cox proportional analysis for factors associated with survival [file ccr-24-0637_supplementary_figure_s9_suppfs9.png]
