# Supplementary material for: Urinary Metabolite Diagnostic and Prognostic Liquid Biopsy Biomarkers of Lung Cancer in Nonsmokers and Tobacco Smokers
Source: Clin Cancer Res. 2024 Jun 5;30(16):3592–602. doi: 10.1158/1078-0432.CCR-24-0637 (PMC11325153; doi:10.1158/1078-0432.CCR-24-0637)
Supplement: Supplementary Table S1 — The diagnostic efficiency of models in non-smoking exploratory and validation cohorts. LCC, Lung cancer cases; PC, Population controls; AUC, area under the curve; CI, confidence interval; SN, Sensitivity; SP, Specificity; NPV, negative predictive values; PPV, positive predictive values; CR, Creatine riboside; NANA- N-acetyl neuraminic acid. [file ccr-24-0637_supplementary_table_s1_suppts1.docx]

| Non-smoker | Exploratory cohort | | | | | | Validation Cohort | | | | |
| --- | --- | --- | --- | --- | --- | --- | --- | --- | --- | --- | --- |
|  | AUC (95% CI) | SN (%) | SP (%) | | PPV (%) | NPV (%) | AUC (95% CI) | SN (%) | SP (%) | PPV (%) | NPV (%) |
| LCC vs PC |  | | | | | | | | | | |
| CR | 0.91 (0.88-0.94) | 70 | 95 | 89 | | 85 | 0.80 (0.74-0.86) | 89 | 60 | 61 | 88 |
| NANA | 0.90 (0.86-0.93) | 84 | 84 | 74 | | 90 | 0.76 (0.69-0.83) | 75 | 72 | 65 | 80 |
| CR + NANA | 0.94 (0.90-0.96) | 80 | 94 | 89 | | 88 | 0.80 (0.74-0.86) | 89 | 61 | 62 | 88 |
| Early stage (I&II) vs PC |  | | | | | | | | | | |
| CR | 0.94 (0.90-0.97) | 92 | 83 | 56 | | 98 | 0.74 (0.65-0.84) | 72 | 77 | 57 | 87 |
| NANA | 0.89 (0.84-0.94) | 80 | 83 | 53 | | 94 | 0.74 (0.66-0.84) | 72 | 75 | 55 | 86 |
| CR + NANA | 0.95 (0.91-0.98) | 90 | 89 | 67 | | 97 | 0.75 (0.66-0.84) | 72 | 77 | 57 | 87 |
| Late stage (III&IV) vs PC |  | | | | | | | | | | |
| CR | 0.90 (0.86-0.95) | 72 | 96 | 86 | | 92 | 0.86 (0.79-0.93) | 79 | 82 | 56 | 93 |
| NANA | 0.91 (0.87-0.95) | 72 | 94 | 76 | | 92 | 0.77 (0.67-0.87) | 76 | 75 | 48 | 91 |
| CR + NANA | 0.93 (0.89-0.97) | 78 | 95 | 82 | | 94 | 0.87 (0.80-0.93) | 79 | 84 | 60 | 93 |
