# Supplementary material for: Urinary Metabolite Diagnostic and Prognostic Liquid Biopsy Biomarkers of Lung Cancer in Nonsmokers and Tobacco Smokers
Source: Clin Cancer Res. 2024 Jun 5;30(16):3592–602. doi: 10.1158/1078-0432.CCR-24-0637 (PMC11325153; doi:10.1158/1078-0432.CCR-24-0637)
Supplement: Supplementary Table S2 — Univariate and multivariate regression analysis for clinical factors, metabolite levels and prognosis in smokers. [file ccr-24-0637_supplementary_table_s2_suppts2.docx]

| **Exploratory cohort** | | | | | | **Univariable** | | | | | | | | | |  | | **Multivariable** | | | | | |
| --- | --- | --- | --- | --- | --- | --- | --- | --- | --- | --- | --- | --- | --- | --- | --- | --- | --- | --- | --- | --- | --- | --- | --- |
| **Factors** | | | **Levels** | | | **N** | | **HR**^1^ | | **95% CI**^1^ | | | **p-value** | | |  | | **HR**^1^ | | **95% CI**^1^ | | | **p-value** |
| **CR + NANA category** | | | Low; High | | | 261 | | 1.70 | | 1.29, 2.22 | | | **<0.001** | | |  | | 1.40 | | 1.06, 1.86 | | | **0.019** |
| **Stage** | | | Early Stage (I & II); Late Stage (III & IV) | | | 257 | | 3.19 | | 2.39, 4.27 | | | **<0.001** | | |  | | 2.89 | | 2.14, 3.89 | | | **<0.001** |
| **Histology** | | | Adenocarcinoma; Squamous cell carcinoma | | | 261 | | 1.43 | | 1.09, 1.88 | | | **0.011** | | |  | | 1.32 | | 1.00, 1.76 | | | **0.05** |
| **Age** | | | n=261 | | | 261 | | 1.04 | | 1.02, 1.06 | | | **<0.001** | | |  | | 1.03 | | 1.01, 1.05 | | | **0.016** |
| **Sex** | | | Male; Female | | | 261 | | 0.88 | | 0.67, 1.15 | | | 0.35 | | |  | | 0.90 | | 0.68, 1.18 | | | 0.4 |
| **Race** | | | African American; European American | | | 261 | | 0.91 | | 0.67, 1.23 | | | 0.54 | | |  | | 0.87 | | 0.63, 1.19 | | | 0.4 |
| **Validation cohort** | | | | **Univariable** | | | | | | | |  | | **Multivariable** | | | | | | |  |  |  |
| **Factors** | | | **Levels** | | **N** | | **HR**^1^ | | **95% CI**^1^ | | **p-value** | | | |  | | **HR**^1^ | | **95% CI**^1^ | | | **p-value** | |
| **CR + NANA category** | | | Low; High | | 206 | | 1.78 | | 1.12, 2.81 | | **0.014** | | | |  | | 1.74 | | 1.08, 2.81 | | | **0.022** | |
| **Stage** | | | Early Stage (I & II); Late Stage (III & IV) | | 206 | | 2.79 | | 1.75, 4.46 | | **<0.001** | | | |  | | 2.23 | | 1.37, 3.63 | | | **0.001** | |
| **Histology** | | | Adenocarcinoma; Squamous cell carcinoma | | 206 | | 1.44 | | 0.85, 2.46 | | 0.19 | | | |  | | 1.14 | | 0.63, 2.08 | | | 0.7 | |
| **Age** | | | n=206 | | 206 | | 0.97 | | 0.95, 0.99 | | **0.011** | | | |  | | 0.97 | | 0.95, 1.00 | | | **0.037** | |
| **Sex** | | | Male; Female | | 206 | | 0.60 | | 0.38, 0.96 | | **0.033** | | | |  | | 0.64 | | 0.39, 1.07 | | | 0.090 | |
| **Race** | | | African American; European American | | 206 | | 0.80 | | 0.32, 1.97 | | 0.62 | | | |  | | 0.95 | | 0.38, 2.35 | | | >0.9 | |
|  |  | ^1^HR = Hazard Ratio, CI = Confidence Interval | | | | | | | | | | | | | | | | | | |  |  |  |
